# Climate values as predictor of climate change perception in the Kingdom of Saudi Arabia

**DOI:** 10.3389/fpsyg.2022.1044697

**Published:** 2022-12-01

**Authors:** Boshra A. Arnout

**Affiliations:** ^1^Department of Psychology, King Khalid University, Abha, Saudi Arabia; ^2^Department of Psychology, Zagazig University, Zagazig, Egypt

**Keywords:** climate change, climate perception, climate values, Self-transcendence values, self-enhancement, hedonic values, egoism, altruism

## Abstract

**Background:**

Understanding public perceptions of climate change and how individuals perceive it is critical to developing effective communication strategies, policies, and socially robust technologies to relieve the risks of climate change. Despite the growing literature on climate change, until now, researchers in Arab countries have not been interested in studying citizens’ perceptions of climate change or identifying the factors that predict it. This study aimed to identify and understand the nature and dynamics of public perceptions of climate change among Arab citizens and detect the level of climate change perception (CCP) and climate values (CV). Also, to detect the predictability of CCP from CV. As well as to reveal the differences between CCP and CV.

**Methods:**

A random sample consisted of 465 participants (236 male and 229 female), residents of the Kingdom of Saudi Arabia; their ages ranged from 30 years and over. The Climate Change Perception Questionnaire (CCPQ) and Climate Values Questionnaire (CVQ) were applied online.

**Results:**

The results found average levels of CCP and CV among the study sample. The results also revealed significant statistical differences in the CCP and CV due to gender in favor of females. As well as, there were significant statistical differences in the CCP due to the career field in favor of agriculture, engineering, and construction workers. Also, there were statistical differences in the emotional subscale of climate perception and CV due to age groups in favor of individuals whose ages ranged from 30 to 45 years. The results also found that the CV were a statistically significant predictor (1.2% of total variance) of climate perception.

**Conclusion:**

The current study showed an average level of CCP and CV among individuals in the Kingdom of Saudi Arabian. The findings also concluded that individuals’ perceptions of climate change are an individual response determined by the individual’s gender, age, and career field and are affected by his values about climate. These findings shed light on the need for climate communications to increase the level of CCP and CV, especially among males and individuals in the age group over 45 years and individuals working in various career fields, whether education, engineering and construction, and commerce and business, etc.; to improve the engagement in mitigation and adaptation measures to climate change.

## Theoretical background

The perception of climate change has grown significantly over the last few decades, while the level of concern has varied through time and worldwide. Though it developed in several Western nations in the late 2000s, skepticism regarding the existence and severity of climate change is mainly low among the general population worldwide (Clarke et al., 2012; Hansen et al., 2012; Altea, 2020).

To encourage widespread public participation and to create successful communication and educational strategies to encourage climate mindfulness and citizenship, it is essential to understand how the general public views climate change. Understanding how people feel about climate change and how it should be addressed is necessary for developing and implementing viable policies and technology that will help us reduce and adapt to it (de Matos Carlos et al., 2020; Fourment et al., 2020; Arnout, 2022a). Various psychological elements, such as information, beliefs, attitudes, anxiety, influence, and perceived threats associated with climate change, have all been called “climate change perceptions (CCP).” The term “perceptions” refers to people’s internal representations of the problem of climate change, including their cognitive (e.g., knowledge), emotional (e.g., feelings), and evaluative (e.g., perceived risks) dimensions. However, we understand that these representations are influenced by social processes and cultural context (Kahan, 2015; Lehner and Stocker, 2015; Clayton and Manning, 2018).

Since the early 1980s, research on public perceptions of climate change has increased. Clayton and Manning (2018) mentioned that surveys have shown that perception and subjective knowledge about climate change have grown during the previous three decades. However, a cross-country study conducted in 2014 in three European nations, China, the United States, and Canada, showed that general respondents were primarily well-informed about climate change (Basannagari and Kala, 2013; Singh et al., 2017; Soliman et al., 2018; Kim and Ahn, 2019).

Climate change perceptions are shaped by cognitive processes (such as information assimilation) and social interaction within a given cultural context. Climate change is recognized primarily through mass communication, interpersonal communication, formal education, and other channels (Sterman and Sweeney, 2007; Spence et al., 2012; Clayton and Manning, 2018). As a result, media like newspapers and television have been essential in shaping public perceptions of climate change, particularly by highlighting debate over the topic and connecting it to pertinent social and political developments (Clayton and Manning, 2018).

According to a qualitative study on climate perceptions, people’s direct associations with climate change are based on recurring themes such as weather changes and potential climate impacts, the causal role of human activities and natural cycles, and the connection between climate change and consumer society. Other studies have revealed that rather than being seen as a standalone problem, the public percept climate change as a component of a larger group of social and environmental problems, including air pollution, industrialization, consumption, and population increase (Milfont, 2012; Spence et al., 2012; Busse and Menzel, 2014).

Consequently, this conceptual link reflects media coverage that frequently discusses climate change in the context of local weather stories, like floods in the UK, as well as a fundamental understanding of nature and natural processes, such as how they are cyclical or continuously changing. It’s also likely that people’s mental associations are brought on by how climate change is perceived as a novel idea about well-known concepts and events, a phenomenon known as “fixation” in the psychosocial literature. This embedded perspective on climate change implies that surveys and interviews will ostensibly reveal “misconceptions” about how various environmental and social issues are related to climate change. Instead of attempting to communicate abstract scientific “facts” that may have little application to everyday life, officials can use such socially rooted narratives to engage the public in climate change actively (Hansen et al., 2012; Gurgiser et al., 2016; Clayton and Manning, 2018; de Matos Carlos et al., 2020).

Numerous studies have shown that people’s views on climate change vary significantly within and within countries. People’s opinions of climate change varied widely around the world, with Latin America and developed Asia displaying relatively high levels of fear compared to other regions. This is probably attributable to the higher risk exposure, cultural values (such as a greater emphasis on environmental protection), political climate (such as left-wing politics), and media coverage style. Studies also show diverse attitudes, from the most engaged and concerned doubters to the ones who are merely apathetic or uninterested (Sánchez-Cortés and Lazos, 2011; Hansen et al., 2012; Lehner and Stocker, 2015; Stoknes, 2015).

Belief and concern about climate change vary according to a range of factors, such as gender and age (specifically, men and the elderly tend to be more skeptical and less concerned about climate change, although the results of studies find that variables of values, an individual’s view of the world and ideology are substantial and statistically significant predictors of demographic, cognitive, or other factors (Daniel et al., 2001; Milfont, 2012; Kahan, 2015; Clayton and Manning, 2018).

Bauman et al. (2018) mentioned that individuals think and behave in line with their endorsed values; this means that if an individual endorses biospheric and altruistic values, his behavior is more pro-environmentally. While the individual who endorse egoistic and hedonic values their behaviors less pro-environmentally.

According to Hicks et al. (2015) about environmental values and the suggested framework by Marshall et al. (2018) about human-environment cultural values, there are two types of environmental values: self-transcendence values contain biospheric and altruistic values; and two self-enhancement values include egoistic and hedonic values (Hicks et al., 2015; Bauman et al., 2018; Marshall et al., 2018). Hicks et al. (2015) found that these environmental values correlated and shaped a consistent pattern in line with human values theory. Marshall et al. (2019) found that environmental value orientations significantly affect the response to climate change, especially biospheric and altruism, while egoism influences grief response. Thus, Marshall et al. (2019) concluded that values might be a valuable communication method for climate change.

The current study aims to reveal the possibility of these values predicting individuals’ perceptions of climate change since the Arab researchers did not focus on studying the psychological explanation of individuals’ perceptions of climate change and what factors contribute to predicting CCP.

## Materials and methods

### Design and participants

This study used a cross-sectional descriptive design to assess the levels of perception of climate change. A comparative design was used to examine CCP and CV in the Kingdom of Saudi Arabia to identify variations in CP and climate values (CV) caused by demographic factors. The researcher selected randomly (465) residents in the Kingdom of Saudi Arabia, divided into subgroups according to demographic variables: gender (male *n* = 236, 50.75%; female *n* = 229, 49.25%), career field (education *n* = 134, 28.82%, commerce, and business *n* = 104, 22.36%, engineering, and construction *n* = 117, 25.16% and agriculture *n* = 110, 23.66%), and also divided to three age subgroups (from 30 to 45-year *n* = 147, 31.61%, 46-to-60-year *n* = 232, 49.90%, and up to 61-year *n* = 86, 18.49%).

### Data collection instrument

The researcher developed the Climate Change Perception Questionnaire (CCPQ) and Climate Values Questionnaire (CVQ) and applied them online using Google Forms. The online link was sent to many individuals in the Kingdom of Saudi Arabia. The CCPQ (Supplementary Appendix 1) consists of 21 items divided into three aspects of CCP: cognitive, emotional, and evaluation. Answers are given using a 5-point Likert questionnaire (never = 1 to very much = 5). And the CVQ (Supplementary Appendix 2) also consisted of 10 items; individuals can answer by choosing one of the 5-point Likert (never = 1 to very much = 5).

### Climate change perception questionnaire validity

Before conducting the exploratory factor analysis (EFA) analysis on the CCPQ, the researcher conducted Bartlett’s test of Sphericity and the Kaiser–Meyer–Olkin measurement of sampling adequacy (KMO) to verify that the sample was adequate for conducting this analysis. Bartlett’s test of Sphericity for CCPQ was significant (χ^2^ = 8,883.753, df = 210, *p* < 0.001), and the KMO value was acceptable at 0.925. CCPQ items’ component load ranged from 0.510 to 0.914. The EFA results suggest that CCPQ consisted of the 3-dimensional construct (Table 1).

**TABLE 1:** Saturations of the items of the climate change perception (CCP) questionnaire by factors after the rotated component matrix.

Items	Emotional	Appraisal	Cognitive
1	0.807		
2	0.913		
3	0.881		
4	0.914		
5	0.853		
6	0.836		
7	0.903		
8	0.867		
9	0.859		
10	0.873		
11		0.817	
12		0.816	
13		0.785	
14		0.827	
15		0.807	
16		0.820	
17		0.769	
18		0.857	
19			0.805
20			0.809
21			0.510
Total	7.979	5.830	1.552
% Of Variance	37.997	27.760	7.390
Cumulative%	37.997	65.757	73.146

Extraction method: Principal component analysis. Rotation method: Varimax with Kaiser normalization.

The CCPQ also exhibits good reliability since Cronbach’s alpha coefficients for the three subscales and total CCP were (0.966, 0.900, 0.70, 0.930, respectively) high. Also, the correlations between the items and the total score of the subscales were high, ranging between 0.540 and 0.924.

### Climate values questionnaire validity

The results of Bartlett’s test of Sphericity and the KMO revealed that the sample was adequate for conducting this analysis. Bartlett’s test of Sphericity for CVQ was significant (χ^2^ = 3,183.952, df = 45, *p* < 0.001), and the KMO value was acceptable at 0.923. CVQ items component load ranged from 0.766 to 0.825. The EFA results showed that CCPQ consisted of the one-dimensional construct (Table 2).

**TABLE 2:** Saturations of the items of the climate values (CV) questionnaire by principal component analysis.

Items	1
1	0.781
2	0.807
3	0.766
4	0.818
5	0.799
6	0.789
7	0.774
8	0.819
9	0.825
10	0.787
Total	6.348
% of Variance	63.477
Cumulative%	63.477

The CVQ also exhibits good reliability since Cronbach’s alpha coefficient was (0.935) high. Also, the correlations between the items and the total score of the subscales were high, ranging between 0.770 and 0.823.

### Data analysis

Using IBM SPSS Statistics, Version 25, the data was statistically examined. The researcher used descriptive statistics, including percentages, frequencies, and the mean, and standard deviation for sociodemographic variables, to gauge how much the study sample perceived and valued climate change. The Kolmogorov–Smirnov test was employed to determine whether the data distribution was normal. The researcher calculated the correlations between scale items and subscales using Pearson’s correlation coefficient. Additionally, Cronbach’s alpha coefficients were utilized to confirm the CCPQ and CVQ’s internal consistency. The factor structure of the CCPQ and CVQ was examined using EFA. Independent samples *T*-test and one-way ANOVA between-groups comparisons were used to detect the differences due to demographic variables, and *p* < 0.05 statistical significance level was approved, and a Scheffe test was used to identify the direction of the differences. Also, a linear regression was applied to examine the predictability of CCP from CV.

## Results

### Climate change perception and climate values scores

To determine the level of CCP and CV, the individuals were classified into three levels of CCP, as follows: low level from (21 to 49), medium level from (50 to 77), and high level from (78 to 105). In the same way, the individuals were classified into three levels of subscales (for example, the emotional subscale of CCP: low level from (10 to 23.33); medium from (23.34 to 36.67); and high level from (36.68 to 50). Also, CV scores are classified into three levels: Low level from 10 to 23.33, medium level from 23.34 to 36.67, and high level from 36.68 to 50. The results in Table 3 indicated an average level of CCP and CV.

**TABLE 3:** Means, standard deviation, and levels of the CCP and CV of the sample study scores.

Measurements	*M*	St. deviation	Level
Emotional	31.077	10.049	Average
Appraisal	25.443	6.759	Average
Cognitive	9.750	2.375	Average
CCP total score	60.759	8.920	Average
CV total score	30.142	9.205	Average

CCP, climate change perception; CV, climate values.

### Means and standard deviation of the climate change perception and climate values and their level

#### Differences in climate change perception and climate values due to demographic variables

The statistical analysis results showed in Table 4 indicated that there were statistically significant differences in CCP total score, emotional subscale, and CV (*t* = 6.364, 2.087, and 2.539, *p* < 0.05, respectively) due to gender. While there were no statistically significant differences in cognitive and evaluation subscales of CCP due to gender (*t* = 0.434 and 0.829, *p* > 0.05, respectively).

**TABLE 4:** Differences in climate change perception and climate values due to gender (male/female) variable.

Measurement	Gender	*N*	Mean	Std. deviation	*t*-test	Sig. (2-tailed)
Emotional	Male	236	30.123	9.302	2.087	0.037
	Female	229	32.061	10.697		
Appraisal	Male	236	25.699	6.116	0.829	0.407
	Female	229	25.179	7.369		
Cognitive	Male	236	9.703	2.398	0.434	0.664
	Female	229	9.799	2.355		
CCP total score	Male	236	55.479	18.595	6.364	0.000
	Female	229	66.201	17.707		
CV total score	Male	236	29.080	9.266	2.539	0.01
	Female	229	31.236	9.32		

There were statistically significant differences in the emotional subscale of CCP and the total score of CCP due to career field. One-way ANOVA was calculated to detect the differences between career field subgroups. The findings in Tables 5, 6 indicated significant statistical differences due to career field in the emotional subscale of CCP and the total score (*F* = 4.456 and 3.935, *p* < 0.05). At the same time, there were no differences in cognitive, evaluation, and CV (*F* = 2.551 and 1.185, *p* > 0.05).

**TABLE 5:** Differences in climate change perception (CCP) and climate values (CV) due to career field variable.

Measurement	Career field subgroups	*N*	Mean	Std. deviation
Emotional	Engineering and construction field	134	31.948	10.308
	Commerce and business field	104	29.952	10.355
	Education field	117	28.957	9.356
	Agriculture field	110	33.336	9.675
	Total	465	31.077	10.049
Cognitive	Engineering and construction field	134	9.955	2.399
	Commerce and business field	104	9.596	2.518
	Education field	117	9.325	2.173
	Agriculture field	110	10.100	2.362
	Total	465	9.750	2.375
Appraisal	Engineering and construction field	134	25.694	7.097
	Commerce and business field	104	25.298	7.567
	Education field	117	24.573	6.347
	Agriculture field	110	26.200	5.882
	Total	465	25.443	6.759
Climate perception	Engineering and construction field	134	65.179	18.177
	Commerce and business field	104	59.615	19.869
	Education field	117	57.376	18.724
	Agriculture field	110	60.054	18.344
	Total	465	60.759	18.920
Climate values	Engineering and construction field	134	29.716	9.012
	Commerce and business field	104	30.173	9.834
	Education field	117	29.932	8.7116
	Agriculture field	110	30.854	9.417
	Total	465	30.142	9.205

**TABLE 6:** Results of group differences in climate change perception (CCP) and climate values (CV) due to a career field variable.

Variables	Groups	Sum of squares	df	Mean square	*F*	Sig.
Emotional	Between groups	1,320.478	3	440.159	4.456	0.004
	Within groups	45,536.735	461	98.778		
	Total	46,857.213	464			
Appraisal	Between groups	42.734	3	14.245	1.185	0.315
	Within groups	2,574.328	461	5.584		
	Total	2,617.062	464			
Cognitive	Between groups	162.292	3	54.097	2.551	0.055
	Within groups	21,040.447	461	45.641		
	Total	21,202.740	464			
CCP total score	Between groups	4,147.581	3	1,382.527	3.935	0.01
	Within groups	161,955.443	461	351.313		
	Total	166,103.024	464			
CV total score	Between groups	85.398	3	28.466	0.334	0.800
	Within groups	39,231.234	461	85.100		
	Total	39,316.632	464			

Significant at 0.05 level.

To determine the direction of these differences, a Scheffe test was applied, and the results are concluded as follows:

The results shown in Table 7 about the direction of the differences between career field subgroups indicated that the emotional subscale of CCP was high among workers in the field of agriculture rather than workers in the field of education (mean difference = 4.379, *p* < 0.05), also the workers in the field of engineering and construction higher than the workers of education field in the total score of CCP (mean difference = 7.803 *p* < 0.05).

**TABLE 7:** Multiple comparisons in the emotional subscale of climate change perception (CCP) and CCP total score due to career field variable.

Measurement	Career field	Agriculture field	Engineering and construction field	Education field
Emotional	Agriculture field	–	4.379*	
	Engineering and construction field	−4.379*	–	
Climate change perception	Engineering and construction field		–	−7.803*
	Education field		7.803*	

*Mean differences were significant (*p*-value < 0.05).

There were differences in the emotional subscale of CCP and CV due to age group. One-way ANOVA was calculated to detect the differences between age subgroups. The findings in Tables 8, 9 indicated significant statistical differences due to age group in the emotional subscale of CCP and CV (*F* = 3.565 and 3.524, *p* < 0.05). At the same time, there were no differences in cognitive, evaluation, and total score of CCP (*F* = 0.253, 1.105, and 1.559, *p* > 0.05).

**TABLE 8:** Differences in climate change perception (CCP) and climate values (CV) due to age group variable.

Measurement	Age subgroups	*N*	Mean	Std. deviation
Emotional	30–45 years	147	32.8503	10.99399
	46–61 years	232	30.0517	9.73443
	Up to 61 years	86	30.8140	8.81778
	Total	465	31.0774	10.04915
Cognitive	30–45 years	147	9.7823	2.22229
	46–61 years	232	9.6164	2.38216
	Up to 61 years	86	10.0581	2.59572
	Total	465	9.7505	2.37491
Appraisal	30–45 years	147	25.6803	7.26880
	46–61 years	232	25.2198	6.78609
	Up to 61 years	86	25.6395	5.76789
	Total	465	25.4430	6.75985
Climate perception	30–45 years	147	63.0204	20.17101
	46–61 years	232	59.8448	17.78861
	Up to 61 years	86	59.3605	19.55079
	Total	465	60.7591	18.92038
Climate values	30–45 years	147	31.3061	9.46704
	46–61 years	232	29.0129	8.99903
	Up to 61 years	86	31.1977	9.03173
	Total	465	30.1419	9.20511

**TABLE 9:** Results of group differences in climate change perception (CCP) and climate values (CV) due to age group variable.

Variables	Groups	Sum of squares	df	Mean square	*F*	Sig.
Emotional	Between groups	712.103	2	356.051	3.565	0.029
	Within groups	46,145.110	462	99.881		
	Total	46,857.213	464			
Appraisal	Between groups	12.461	2	6.231	0.253	0.777
	Within groups	2,604.601	462	5.638		
	Total	2,617.062	464			
Cognitive	Between groups	23.153	2	11.576	1.105	0.332
	Within groups	21,179.587	462	45.843		
	Total	21,202.740	464			
CCP total score	Between groups	1,113.846	2	556.923	1.559	0.211
	Within groups	164,989.178	462	357.119		
	Total	166,103.024	464			
CV total score	Between groups	590.807	2	295.404	3.524	0.030
	Within groups	38,725.825	462	83.822		
	Total	39,316.632	464			

Significant at 0.05 level.

To determine the direction of these differences, a Scheffe test was applied, and the results are concluded as follows:

The results shown in Table 10 about the direction of the differences between age subgroups indicated that the emotional subscale of CCP and CV were high among individuals whose ages ranged from 30 to 45 subgroup rather than 46–60 years old subgroup (mean difference = 2.799 and 2.92, *p* < 0.05, respectively).

**TABLE 10:** Multiple comparisons in the emotional subscale of climate change perception (CCP) and climate values (CV) due to age group variable.

Measurement	Age subgroups	30–45 years	46–61 years
Emotional	30–45 years	–	2.799*
	46–61 years	−2.799*	–
Climate values	30–45 years	–	2.292*
	46–61 years	−2.292*	–

*Mean differences were significant (*p*-value < 0.05).

#### Predict climate change perception from climate values

The researcher used a simple linear regression to detect the ability to predict CCP from CV. The simple regression model predicting CCP total score proved to be statistically significant (*F* = 5.392, *p* < 0.021). The results in Table 11 indicated that the CV could predict CCP statistically significant; CV total score explains 1.2% of the variance in total CCP (Beta [β] = 0.107; 95% Confidence Interval: [18.080, 2.322]; *p* < 0.05). We can predict the CCP total score from this prediction equation: CCP = 54.112 + 0.221× CV.

**TABLE 11:** Results of regression analysis of climate values (CV) on climate change perception (CCP).

Model	*R* ^2^	Durbin–Watson	*F*	Independent variables	Unstandardized coefficients		Standardized coefficient	*t*	Sig.
					B	SE	Beta		
1	0.012	0.805	5.392	Constant	54.112	2.993		18.080	0.000
				Climate values	0.221	0.095	0.107	2.322	0.021

## Discussion

The study findings showed an average level of CCP (total score and subscales). The result of the current study is consistent with the findings of the study by Hussein et al. (2019) that only a small percentage of citizens in Lebanon have a high level of awareness of climate change and their intentions to reduce the carbon lifestyle. Clayton and Manning (2018) stated individuals percept climate change as part of a broader set of social and environmental problems, such as air pollution, industrialization, consumption, and population increase, rather than as a stand-alone problem. Therefore, the study sample’s perception of climate change was average. Also, the results found an average level of climate value. This finding can be interpreted as weather events are not percept as evidence of climate change unless one believes in climate change. Therefore, the level of CCP and CV were average among the study sample. Because, in general, climate change is viewed as an issue of low importance to all other issues and challenges facing society, such as economic development, global political instability, public health, and other environmental problems. As well as the deep cultural conflict that exists over this issue (Sterman and Sweeney, 2007).

Gifford (2011) hypothesized that 30 factors might act as barriers to individuals’ perceptions of climate change and changing their behaviors, including limited awareness, old brain, ignorance, environmental numbness, mistrust, reduced judgment of risk, unrealistic/biased optimism, and lack of perceived behavioral control, ideology, superhuman powers, technological revolution, others of importance, social norms, social comparison, perceived injustice, behavioral momentum, the strength of habits, resistance, and denial.

These results about the average level of perception of climate change and CV can interpret using the framework of values of Schwartz (1994), as it has been shown in many studies that support the values of the “transcendent self,”—which includes a focus on protecting and caring for others—is linked to acceptance of the reality of climate change, and level of concern about the issue, as well as support for policies designed to relief climate change and a willingness to act in an environmentally friendly manner. Drawing on an alternative concept of the “Schwartz” framework, others have suggested the importance of other-oriented values such as selfishness, altruism, and the biosphere as negative influences on climate change recognition, policy support, and pro-environmental behavior. Selfishness negatively affects recognition of the problem of climate change and support for policies designed to address climate change, while altruism and biosphere influence positively. Studies showed that other-oriented values (such as altruism) are associated with environmental concerns (including climate change) and support for climate change policies and pro-environmental actions.

These findings are also due to the role of the media as the primary source of public information on climate change. Unsurprisingly, the media play a crucial role in shaping public attitudes, responses, and perceptions of climate change. The study by Carmichael and Brulle (2017) showed a clear impact of media on public anxiety about climate change and their perception of it. Clayton and Manning (2018) argue that humans live in a bubble of self-deception and a false sense that climate change is a problem for the future rather than an urgent crisis that must be addressed to secure life on Earth.

These findings can also be explained in terms of the perception of climate change through construal level theory (CLT), which indicates that objects, events, and structures can be thought of in somewhat abstract terms depending on the psychological distance between them. CLT theory showed a relationship between psychological distance and people’s response to a particular event. Psychological distance consists of four dimensions: spatial, social, temporal, and virtual. Each dimension is related to the others, although there are no common denominators. Psychological distance is one of the main psychological structures that explain the more realistic or abstract perception of objects and events around people. An event is viewed as near or far from a psychological point of view. When viewed as psychologically close, the representation is more realistic, while the representation is more abstract when viewed as psychologically distant. Thus, people perceive climate change more realistically when they see it up close; and as a result, there may be an increased willingness to engage in pro-environmental and resilient behaviors. When people have a more abstract representation of the event, climate change is seen as far from occurring. We can conclude that psychological distance determines an individual’s perception of climate change. The results of studies such (Spence et al., 2012; Busse and Menzel, 2014; Carmi and Kimhi, 2015; Jones et al., 2017; Singh et al., 2017; Soliman et al., 2018; Kim and Ahn, 2019; Kyselá et al., 2019) found an effect of psychological distance on individuals’ perception of climate change and engagement in pro-environmental behaviors.

From a cognitive psychology perspective, the perception of climate change refers to how we think and how the brain processes information. Perception is an individual process determined in light of the personality traits and the habits and traditions of the society in which he lives. The perception process is affected by many situational and personal factors, and the individual’s attitudes may effectively impact his perception of stimuli. The attitudes of individuals toward climate change consist of three main components: a cognitive component that includes ideas, knowledge, and beliefs that stem from memory when paying attention to the issue of climate change, an emotional component, which refers to the feeling associated with an object or event, and then a behavioral component, includes the type of action or readiness for behavior inherent in the situation. The attitude toward climate change is strong and consistent if these three components are compatible. According to the strength of the emotional component toward the issue of climate change, the strength and quality of our actions and responses are determined by whether we will make an effort to combat climate change or will we fail to act and not seek to make any effort, but instead deny the issue in its entirety and fight its supporters. In light of this, the individual’s perception and values about climatic changes are determined. Hence, individuals differ in their awareness of climate change and their CV according to their characteristics, customs, and traditions of society and their attitudes toward climate change.

Clayton and Manning (2018) reported that perception of climate change varies according to various factors, such as gender and age (specifically, men and older people tend to be more aware and less anxious about climate change). However, studies find that variables of values, an individual’s view of the world, and ideology are strong and statistically significant predictors of CCP rather than demographic, cognitive, or other factors. Also, the results found differences between males and females in the emotional subscale of CCP, the total score of CCP, and CV in the favorite of females. This result is due to females’ lack of social and economic power; they tend to have fewer economic resources than men. Also, females find it difficult to access assistance services in disasters such as climate change. Thus, females are more aware of climate change and have higher CV and participation in mitigation measures.

Results also revealed significant statistical differences due to the career field in the emotional subscale of CCP and total score of CCP in favorite of workers in the agriculture and engineering and construction fields rather than career workers field subgroups. Clayton and Manning (2018) mentioned that societies whose livelihoods are directly linked to local climatic conditions, such as farmers in developing countries, may recognize climate change closer. Basannagari and Kala (2013) provides evidence that apple farmers in the Indian Himalayas percept climate change as having led to modifications in their farm use practices, delays in harvest periods, and adverse effects on fruit quality. Also, in Mexico, Sánchez-Cortés and Lazos (2011) similarly found that in addition to being aware of changes in rainfall and temperature, farmers responded to these changing conditions by introducing the seeding season for corn and planting new crops.

The findings also found differences between age subgroups in the emotional subscale of CCP and CV in favorite of individuals in the 30–45-year subgroup. This difference is because, according to the ages of individuals, their attitudes toward climate change differ from acknowledgment or denial of the occurrence of climate change to either interest in combating climate change or denial and resistance. In light of this, the individual’s perception and values regarding climate change differ. The age subgroup from 30 to 45 years old, we find their attitude toward environmental issues stronger and open to weather news around the world and good readers of environmental issues, climate and global agreements on climate change and integrated into daily life with its problems to a greater degree than the elderly who are gradually isolated and do not care about news about the world but instead they may not care about the environment and climate issues. Weber (2016) stated that men are more skeptical of climate change than women and that younger adults have demonstrated stronger attitudes toward the environment and climate than older adults.

The differences in CCP and CV are compatible with the results of several studies that showed that individuals are highly heterogeneous in their perception of climate change—both within and between countries (Arnout, 2022b). There is a wide variation in people’s perceptions of climate change, with Latin America and developed countries in Asia showing relatively high levels of anxiety compared to other parts of the world. This is likely due to increased exposure to risks, cultural values, political context, and the nature of media coverage. Studies also point to distinct sets of attitudes, ranging from the most active and concerned about climate change to the uninterested or passive deniers.

In light of the theory of the five D barriers developed by Stoknes (2015), we can explain the differences between CCP and CV in the study sample. These five D barriers are Distance, Doom, Dissonance, Denial, and iDentity; they are interconnected and act as invisible barriers within the individual that prevent them from perceiving climate change or engaging in mitigation measures and pro-environmental behavior. These 5 D barrier levels differed between individuals; hence, their levels of CCP and CV differ. Kahan (2015) added that individuals have a strong incentive to form perceptions of risks in general, including those related to climate change, that support their overall sense of who they are, that is, that reinforces their identity. Since our identities are closely linked to our social groups and our preferences for how society is organized, the drive to protect one’s identity can lead to different groups of people coming to percept particular risks or issues, such as climate change, in very different ways.

Also, the results of the current study indicated that the CV have statistically significant predictability of CCP. This result is in line with the study of Marshall et al. (2019), which found that environmental value orientations influenced the individual response to climate change. Ajzen (2005, 2012) mentioned that people form their attitudes toward events (such as climate change) through their fundamental values and beliefs. Therefore, values predict individuals’ perceptions of climate change, attitudes toward this issue, and the decisions that lead to behavior. Daniel et al. (2001) reported that values are essential in building an individual’s personality and affect his attitudes, decisions, and behaviors. Ajzen (2012) refereed those values raise the individual’s awareness of himself and the climatic changes that occur around him and adapt to them with free will and mindfulness. Through the values that the individual adopts, he will be able to face all challenges in his life, deal with crises, and urge him to think about them (including the climate change crisis).

Many theories explain the role of values in perception, attitude, and behavior. Values and beliefs (VBN) theory has explained that environmental behavior is the indirect result of following an individual’s deeply held values, usually, those that demonstrate an interest beyond the individual’s immediate self-interest and include altruism toward other human beings as altruism toward the environment. Theories of values have been developed to influence global views about the relationship between humans and nature, influencing specific beliefs about the consequences of environmental problems and actions, including climate change. The theory of planning behavior (TPB) also suggests that intentions engage in behavior result from three factors: attitudes about the behavior, subjective norms, and perceived control. Each of these factors, in turn, is influenced by the specific beliefs of the individual. Attitudes are formed in response to beliefs about the consequences of behavior. The TPB theory of planned behavior successfully explains climate-related intentions and behavior variations.

## Conclusion

The current study showed an average level of CCP and CV among individuals in the Kingdom of Saudi Arabian. As well as the current study concluded that individuals’ perceptions of climate change are an individual response determined by the individual’s gender, age, and career field and is affected by his values about climate. Individuals live in the same extreme climatic conditions but differ in their perception of them according to those determinants. The results found statistically significant differences in the emotional subscale of CCP, total score, and CV due to gender and age group variables in the favorite of females and individuals whose ages ranged from 30 to 45-year subgroups. Also, the results found statistically significant differences in the CV due to career field variables in favor of agriculture, engineering, and construction fields. As well as the results revealed that the climate value represents a statistically significant predictor of CCP. These findings shed light on the need for a climate communications system to increase the level of CCP and CV, especially among males and individuals in the age group over 45 years, and individuals working in various professions, whether education, business, and others; to improve the engagement in mitigation and adaptation measures to climate changes that are currently taking place in the Kingdom of Saudi Arabia and other Arab countries from extreme changes in temperature, rain, floods, and others.

### Strengths, limitations, and future directions

This study is a predictive, descriptive study to measure CCP and CV levels in the Kingdom of Saudi Arabia and predict CCP from CV. One of the strengths of this study is that it is considered one of the first Arabic studies that examine CCP and CV. Also, the current study is considered a comparative study of the CCP and CV levels due to gender, career field, and age group variables.

Among the limitations of this study include a predictive, descriptive comparative research design; thus, we need more intervention studies to examine how we can improve CCP and values.

## Data availability statement

The original contributions presented in this study are included in the article/Supplementary material, further inquiries can be directed to the corresponding author.

## Ethics statement

The studies involving human participants were reviewed and approved by the KKU (EDU-29/1443/10). The patients/participants provided their written informed consent to participate in this study.

## Author contributions

The author confirms being the sole contributor of this work and has approved it for publication.
