# Supplementary material for: Climate values as predictor of climate change perception in the Kingdom of Saudi Arabia
Source: Front Psychol. 2022 Dec 1;13:1044697. doi: 10.3389/fpsyg.2022.1044697 (PMC9751924; doi:10.3389/fpsyg.2022.1044697)
Supplement: Supplementary file 1 [file Data_Sheet_1.doc]

**Appendices:**

**Appendix 1.**

**Climate change perception questionnaire (CCPQ):**

| 1. I get upset when I hear about climate disasters. |
| --- |
| 1. I am in solidarity with the associations for preserving the climate. |
| 1. I am angry at the behavior of some individuals that harm the environment. |
| 1. I feel sad when I hear dangerous weather news around the world. |
| 1. It saddens me that the place I live in has been affected by climate change. |
| 1. I fear failing to take precautionary measures against catastrophic climate changes. |
| 1. I feel sad because of climate change's impact on nations' economies. |
| 1. I am frightened by the increasing incidence of environmental disasters around me. |
| 1. I am concerned that others talk about the impact of climate change on human health. |
| 1. Because of my fears, I make sure to buy magazines and books on the climate problem. |
| 1. I believe that climate change is real and its effects are noticed everywhere. |
| 1. I am interested in climate news. |
| 1. I follow programs that are concerned with the weather. |
| 1. I know the harmful effects of climate change on my health. |
| 1. I believe that human behavior contributes greatly to climate change. |
| 1. I participate in providing assistance services to individuals affected by climate change. |
| 1. Individuals must adhere to pro-environmental behaviour. |
| 1. I am against those who reject the existence of climate change. |
| 1. I believe that climate change is a catastrophe for the planet. |
| 1. It is religiosity to adhere to behaviors that do not increase the problem of climate change. |
| 1. I regret that some people deny the issue of climate change. |

**Appendix 2.**

**Climate values questionnaire (CVQ):**

| 1. I will do my best to reduce my daily activities that increase global warming. |
| --- |
| 2. Being environmentally conscious enhances our quality of life and well-being. |
| 3. We must preserve the beauty of nature for all to enjoy. |
| 4. I must personally commit to mitigating behaviors on global climate change. |
| 5. Preserving the environment is part of my identity. |
| 6. I am responsible for maintaining the framework I live in (water, air, soil). |
| 7. I'm giving up some of my favorite personal activities to protect the environment. |
| 8. We must cooperate to make our environment safe and live happily. |
| 9. Man's fate is linked to planet Earth's biological balance. |
| 10. It is wrong for the individual to stop behaviors he loves to reduce energy consumption and mitigate the problem of climate change. |
